# Syphilis Notifications and the Triggering Processes for Vertical Transmission: A Cross-Sectional Study

**DOI:** 10.3390/ijerph17030984

**Published:** 2020-02-05

**Authors:** Samara Isabela Maia de Oliveira, Cecília Olívia Paraguai de Oliveira Saraiva, Débora Feitosa de França, Marcos Antônio Ferreira Júnior, Libna Helen de Melo Lima, Nilba Lima de Souza

**Affiliations:** 1 Department of Nursing, Post-Graduate Program in Nursing, Federal University of Rio Grande do Norte, Natal 59072-970, Brazil; cecilia_olivia@yahoo.com.br (C.O.P.d.O.S.); deborafranca_enf@yahoo.com.br (D.F.d.F.); marcos_nurse@hotmail.com (M.A.F.J.); libnahelengmr@gmail.com (L.H.d.M.L.); nilba.lima@hotmail.com (N.L.d.S.); 2 Department of Nursing, Integrated Institute of Health of Federal University of Mato Grosso do Sul, Federal University of Mato Grosso do Sul, Campo Grande 79070-900, Brazil; 3 Department of Nursing, Federal University of Rio Grande do Norte, Natal 59072-970, Brazil

**Keywords:** congenital syphilis, vertical transmission of infectious disease, prenatal care, public health surveillance, epidemiology, geographic mapping

## Abstract

Syphilis is a disease that is found all over the world that causes damaging effects to the fetus through vertical transmission. This study aimed to analyze the processes that trigger the vertical transmission of syphilis through gestational and congenital syphilis notifications. It is a cross-sectional study. The sample totaled 129 notifications of syphilis in pregnant women and 132 notifications of congenital syphilis in the city of Natal, from 2011 to 2015. Data were obtained from the Information System for Disease Notification. The Chi-square, Student’s and Fisher’s tests were used to verify associations of interest. Diagnosis of maternal syphilis was predominant in the third trimester of pregnancy. Only 1.6% of the pregnant women were registered with an adequate treatment regimen, of these 16.3% had the concomitant treatment with their partners. Of the affected children, 78.8% were registered as asymptomatic. The factors that trigger vertical transmission are related to the late diagnosis of the pregnant woman and sexual partner(s) and the deficiencies in clinical/therapeutic management in relation to the phase of the disease. Strategies of professional training should be adopted to notify and expand the provision of information for epidemiological surveillance, aiming to strengthen care, reduce vertical transmission and enable the continuous analysis of this problem.

## 1. Introduction

Syphilis is a major health problem which can be found worldwide, especially in underdeveloped countries. Among its damaging effects are the gestational and obstetric risks present in the occurrence of vertical transmission [1,2]. Adverse outcomes for the pregnant woman and the child are represented by miscarriage, premature birth, neonatal death, low birth weight, and birth of neonates with clinical evidence of infection [3].

Congenital syphilis (CS) is a preventable condition through the control of cases of acquired syphilis in women and their sexual partner(s) during family planning or prenatal care. Moreover, the failure to perform the actions recommended by the relevant health authorities contributes to an increase in the cases of CS and assumes at each event the role of triggering the occurrence of the illness.

The rate of congenital syphilis in Brazil was 6.8 per 1000 live births and in pregnant women 12.4, between 2010 and 2016. Syphilis is characterized as a condition of compulsory notification, in 2015 there was an increase of notifications in cases of pregnant women in all regions of Brazil. Meanwhile, the northeast region showed a decrease of 1%, below the national average [4].

In the context of the Northeastern Brazilian states, the incidence of CS per 1000 live births in 2013 was highest in the states of Sergipe (11.2), Alagoas, and Ceará (7.7), Pernambuco (7.1) and Rio Grande do Norte (5.9). It is noteworthy that, compared to the incidence per thousand live births for syphilis in pregnant women (SPW) in the respective federal units—Sergipe (8.2); Alagoas (4.1); Ceará (5.3); Pernambuco (5.1) and Rio Grande do Norte (3.6)—there is a variation in reporting for syphilis types, which is related to underreporting of the disease [5].

It is also noted that notifications of syphilis in pregnant women (SPW) were lower than those of CS in most northeastern states, which does not coincide with the reality of other regions of Brazil [4]. This denotes a deficiency in prenatal care for the early diagnosis and notification of these pregnant women, a determinant factor when questioning the quality of prenatal care [6].

In the state of Rio Grande do Norte, Brazil, a study [7] on notified cases of CS between April 2009 and April 2014, reported a growth in reports each year, with a total of 684 cases during the period. The concentration of the notifications was in the capital, Natal, with a percentage of 49.85% of the state, which demonstrates a greater movement of CS records in the location [7]. Thus, the investigation area in this study was the city of Natal, due to the representation of the municipality in the notification rates for congenital syphilis in the state of Rio Grande do Norte.

It is relevant for the control of syphilis at local and national levels to know the factors that compromise the eradication of the disease. Thus, investigations focused on the factors involved in syphilis transmission are essential for the development of measures that reduce the incidence of the disease in pregnancy and minimize the risks of vertical transmission.

In this respect, the efficient performance of epidemiological surveillance managers and healthcare professionals in individually analyzing each notified case of maternal and congenital syphilis is highlighted, in order to accurately record the occurrences that culminated in vertical transmission. These records highlight the reality of the disease in the research field and allow the development and implementation of action strategies to control the disease.

Based on this, the present study sought to investigate which factors recorded in the notification forms are regarded as triggers for vertical transmission of syphilis? Therefore, this study aimed to analyze the processes that trigger the vertical transmission of syphilis through gestational and congenital syphilis notifications. 

## 2. Materials and Methods 

### 2.1. Study Design and Context

This is a cross-sectional quantitative approach to epidemiological research based on secondary data, which investigated the triggering factors for the occurrence of congenital syphilis, determined from the analysis of the data reported in the files from the Information System for Disease Notification (ISDN). The research area was the city of Natal, due to the municipality’s representativeness in the notification rates for congenital syphilis in the state of Rio Grande do Norte [8]. 

### 2.2. Participants 

Data from the records of women notified as having gestational syphilis, as well as children with congenital syphilis, were included in the study. Records of pregnant women with syphilis although without notification of congenital syphilis, were excluded, due to the need to investigate the concomitant factors related to cases of vertical transmission. Children with congenital syphilis from mothers who did not receive prenatal care were excluded from the study, as well as cases with duplicate reporting, incomplete data or inconclusive reports.

### 2.3. Data Collection Procedures 

The sample selection was made from the comparison of the notification banks, in order to select the cases of syphilis in pregnant women that resulted in vertical transmission. For this purpose, the year of notification of syphilis cases, the date of birth of the child, the presence of the same maternal name both in the pregnant individual’s syphilis file and in the congenital syphilis file were used as comparison criteria. The notifications eligible for the study corresponded to cases registered between June 2011 and December 2015.

ISDN databases tabulated in the Microsoft Excel 2010 software were filtered for these selection criteria and a total was found of 129 notifications for gestational syphilis and 132 notifications for congenital syphilis. These surplus cases for congenital syphilis refer to the occurrence of two twin pregnancies. Data were collected between May and August 2016. 

### 2.4. Variable Definition 

The analysis involved two dependent variables: children with congenital syphilis and pregnant women with syphilis. The dependent variable of interest in this study is children affected by vertical transmission of the disease. However, to identify the mechanisms that triggered mother-to-child transmission, it is necessary to understand the factors that influenced the continuity of syphilis during pregnancy. Thus, it was assumed that a pregnant woman with syphilis is also a dependent variable of this study. These dichotomous variables were obtained after the mother and child performed the Rapid Syphilis Test using non-treponemal tests, with treponemal serological confirmation, or clinical signs of the disease. Independent variables refer to factors associated with the occurrence of vertical transmission, namely: syphilis diagnostic process; treatment of the pregnant woman; and treatment of partner(s). Regarding the outcome of congenital syphilis, the clinical aspects of the child are variables. 

### 2.5. Statistical Analysis 

Microsoft Excel 2010 software tables generated from the full tabulation of data from the notification files were used as collection instruments. These were submitted to a descriptive analysis and dispersion measurements in order to obtain an overview of the results found. To achieve this, the qualitative variables were initially analyzed from the absolute and relative frequency. Regarding quantitative variables, measurements of central tendency, mean and dispersion analysis with standard deviation were used. 

Chi-square and Fisher’s tests were used to determine the association between sociodemographic variables (age, race and educational level) and the period of diagnosis of maternal syphilis. Student’s *t*-test evaluated the difference between the mean titration of the maternal Venereal Disease Research Laboratory (VDRL) and the titration of the child’s non-treponemal test according to signs or symptoms. 

For geo-referencing analysis, the Terraview software was used to identify spatially how the behavior of syphilis occurred at the research site. For statistical analysis and the generation of geo-referencing thematic maps, the following software were used: Statistical Package For Social Sciences (SPSS) version 20 (IBM, Armonk, NY, USA), R version 2.12 (R Foundation for Statistical Computing, Vienna, Austria) and Terraview version 3.4.0 software (National Institute for Space Research, São José dos Campos, Brazil). The present study is the result of a master’s thesis entitled: Notifications of gestational and congenital syphilis: an epidemiological analysis.

### 2.6. Ethical Considerations 

The research protocol received was approved by the Ethics Committee of the Federal University of Rio Grande do Norte, under number 1,449,134, with a Certificate of Presentation for Ethical Appreciation 53305315.3.0000.5537. 

## 3. Outcomes 

A total of 129 notifications of syphilis in pregnant women and 132 of congenital syphilis were identified, which allowed us to identify an attack rate corresponding to 56.8% and signaled a high probability of pregnant women with syphilis causing vertical transmission in the period studied. 

Regarding the temporal analysis, there was an increase in the number of cases registered in 2012 (SPW 38% and CS 37.1%) and a lower number in 2014 (SPW 13.2% and CS 10.6%), which demonstrates a drop in the number of notifications. In 2015 (SPW 13.2% and CS 20.5%), there was a divergence in the number of cases, where CS cases were higher than SPW. 

The maternal profile identified in this study revealed a majority of pregnant women aged between 21 and 35 years, with an average of 24.78 years, with a Standard Deviation (SD) of approximately 7.12, which converges with the sexual and reproductive age range of the woman. Regarding race, they were characterized as mixed race (70.5%) and were predominantly residents of the urban area (95.3%) of the municipal of Natal (97.7%). 

Although the field of education had a significant lack of under-registration (31.0%), with some left in blank (11.6%) in regard to the completion of notification files, the consolidated data portrayed a low education profile, with a noticeable complete or incomplete elementary school education corresponding to 48.8% of cases. Regarding maternal occupation, 65.2% were registered as housewives. 

Prenatal care stood out as the main period for the diagnosis of syphilis (55.8%); however, the number of cases with late detection records is significant, with 39.5% of women diagnosed at the moment of birth and 3.9% only after birth. 

Regarding laboratory detection data, the reagent non-treponemal test was performed in 115 cases, but it was not performed for ten investigated women, which shows a loss of diagnostic opportunity. This fact corroborates with the data from the confirmatory treponemal test, which presented 87 cases related to non achievement, as shown in Table 1. 

The gestational period of the notification suggests that this was the moment when the woman was diagnosed with syphilis. In this way, the third trimester stands out as the main period of notification, which is associated with syphilitic infection during the gestational period, or can even indicate a significant delay between the moment of diagnosis and notification. 

In regard to the clinical characterization of the pregnant women, they were mostly registered as primary syphilis (51.2%). Although the highest percentage presents VDRL titration between 1 and 8 (39.5%), unexpected titles for this clinical form, the possibility of signs of evidence for primary infection rather than titration should be considered, as well as the historical exposure to the syphilis identified in the diagnostic evaluation.

Regarding the inferential analysis, the results of the Chi-square and Fisher’s tests, at the significance level = 5%, identified that there is no evidence of association between sociodemographic variables (age, race and educational level) and the period of diagnosis of maternal syphilis, according to Table 2.

Regarding the diagnostic tests for syphilis in pregnant women at delivery/curettage described in the congenital syphilis notification file, they point to the presence of reagent non-treponemal tests (94.6%) with accumulated titrations (57.4%) in the range of 1 to 8. 

By analyzing the maternal diagnosis, which was mostly performed during prenatal care, when comparing the results of the reagent non-treponemal test at the time of delivery, the data suggest a possible therapeutic failure in the follow-up of the pregnant woman or reinfection during pregnancy. 

The treatment regimen prescribed to the partner (10.9%) and the pregnant woman (42.6%) emphasized Penicillin G benzathine 7,200,000 IU. This regimen is prescribed for the tertiary clinical phase or for late latent syphilis with a duration of longer than one year. However, we highlight in this analysis the number of partners (38%) who did not receive the treatment or did not do it concomitantly with the pregnant woman (48.1%). 

Of the sample studied, 3.1% of pregnant women and 0.8% of partners used another drug regimen to treat syphilis. Despite being a smaller quantity compared to the group, this data interferes with the adequacy of maternal treatment. This fact may be linked to the shortage of penicillin in Brazil and throughout the world in recent years. 

Thus, this information corroborates with the inadequate treatment of pregnant women (76.7%), being that this criterion meets the appropriate treatment according to the clinical phase of syphilis, the treatment of pregnant women with penicillin, and the treatment of the partner simultaneously with the woman. 

Among the categories that were emphasized for the reason of non-treatment of the partner, the most noteworthy is that they were no longer in contact with the pregnant woman (41.7%). The data also show the low diagnostic adherence, due to the difficulty of actively finding the cases, which corroborates with another relevant category in this analysis, the absence of the diagnostic confirmation of the partner (19.4%). 

Regarding the outcomes of vertical transmission, the data show VDRL titrations between 1 and 8 (54.5%), titles also identified in most pregnant women. The predominance of children registered as asymptomatic (78.8%) is also noteworthy. 

Table 3 presents the results of the application of the Student’s *t*-test to assess the existence of differences between the mean of the titration of the maternal VDRL and the titration of the non-treponemal test of the newborn according to signs or symptoms. Considering a significance level of 5%, evidence was found that there is a difference between the mean titration of the maternal VDRL and of the child according to signs and symptoms.

It is noteworthy that one case of congenital syphilis resulted in death. Thus, considering the verification of the rate indicator for cases of fatality for this group, a rate of 0.75% was detected. 

Regarding the follow-up care of children with syphilis, 94.7% underwent treatment. Of these, 69.5% used Penicillin G Crystalline 100,000 to 150,000 IU/kg/day for 10 days and 25.2% another type of therapeutic regimen. We highlight the higher concentration of records related to the lack of implementation of the non-treponemal cerebrospinal fluid test (73.5%), as well as the non-implementation of an X-ray of the long bones (47%). These data demonstrate possible failures in the follow-up of child care in regard to actions to combat the disease and identification of possible sequelae. 

In the category “Treatment of the mother/pregnant woman”, the item “partner treated concurrently with the pregnant woman” presented 43 (33.3%) ignored cases, which is important data that corroborates with the correct treatment of the pregnant woman, helping in a reduction in the risk of retransmission. 

In this analysis, the ascending titration field is highlighted, which refers to the follow-up of VDRL titles in the outpatient follow-up of the child. Although 87.1% of the records were completed, 73.5% were referred to as unrealized.

Regarding the investigation aspects of the illness, in the item “prenatal health unit”, it was observed that 71.9% of the notifications were incomplete, which makes it difficult to verify the care provided to pregnant women during the entire period of pregnancy. Regarding management, the absence of this data makes it impossible to identify health units that need management support to control syphilis, such as professional training and the sending of supplies. 

For data relating to the notifying unit, it was possible to detect the concentration of syphilis notifications in pregnant women in regard to the level of care, in which 69.8% were performed in hospital units and 30.2% were registered from Family Health Units (FHU). 

**Figure 1:**
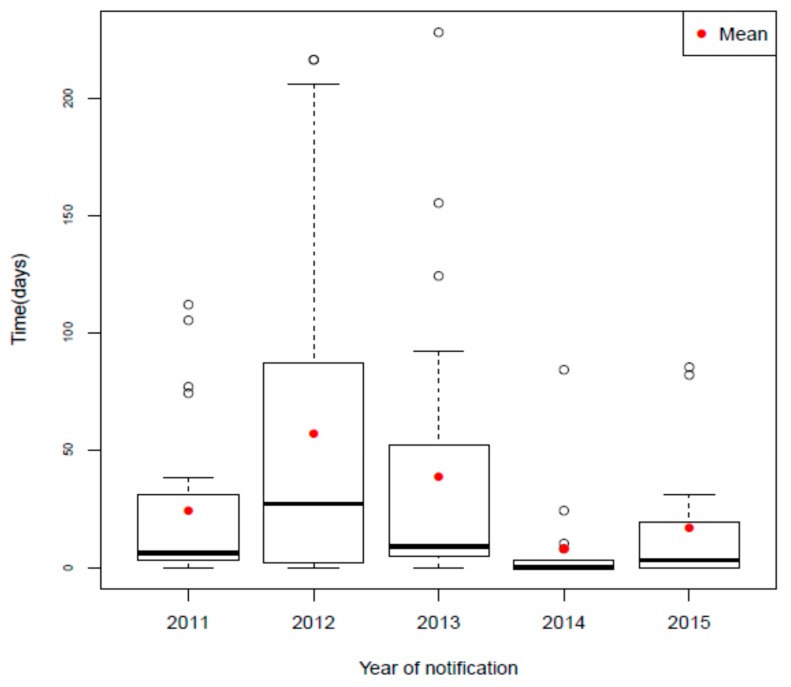
Time between diagnosis and notification of 129 cases of gestational syphilis in the municipality of Natal, Rio Grande do Norte, Brazil, from 2011 to 2015.

Comparing the time between the day of diagnosis and the day of notification, there is a greater variation in the mean of days for notification of syphilis in pregnant women, as shown in Figure 1. 

The figure illustrates a dispersion in the number of days between diagnosis and notification of the pregnant woman, with atypical points present in all the years shown. The year 2012 stands out with the highest mean number of days and 2014 shows a progression close to seven days, as recommended by the Ministry of Health. 

Regarding the interval between the diagnosis and notification of congenital syphilis, a smaller variation was observed between the means, which are close to the compliance of the compulsory weekly notification attributed to syphilis cases. 

These results suggest better epidemiological surveillance processes at the hospital level, at pediatric outpatient clinics or delivery centers and in the reporting of cases of vertical transmission. This fact may be related to the presence of the structured Hospital Epidemiological Surveillance Nucleus (HESN) in the services, which impacts on the processes of disease surveillance. 

**Figure 2:**
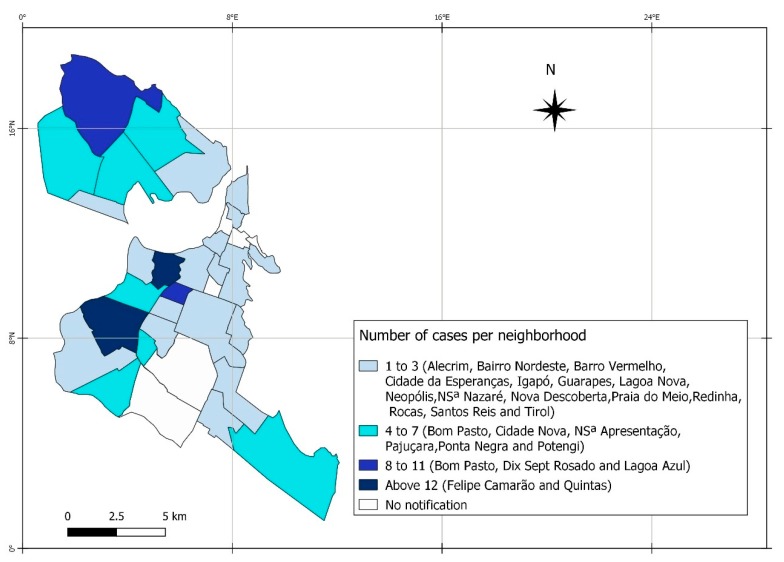
Distribution of 129 reported cases for gestational syphilis from 2011 to 2015 in Natal, Rio Grande do Norte, Brazil.

The main locations of residence where pregnant women were affected most by syphilis are the neighborhoods of Felipe Camarão and Quintas, both supported by the Western Sanitary District of the investigated municipality, as shown in Figure 2. 

**Figure 3:**
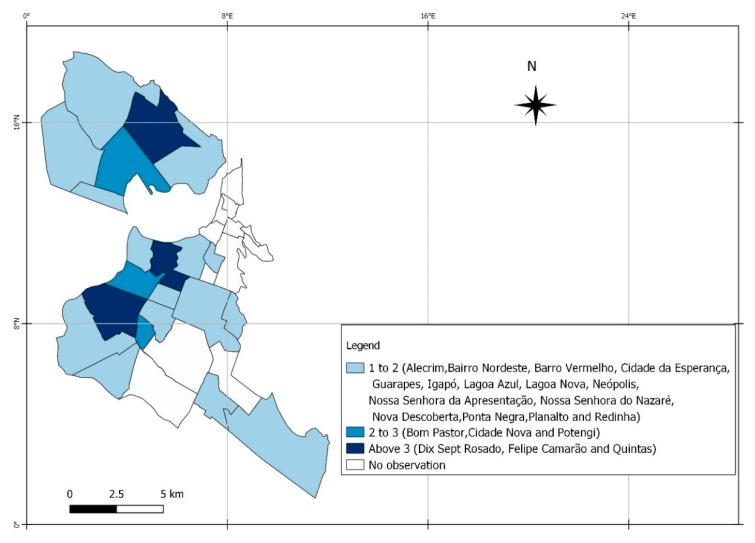
Distribution of partners who were not treated concurrently with pregnant women by location of residence in the municipality of Natal, Rio Grande do Norte, Brazil, from 2011 to 2015. A total of 129 pregnant women.

The main locations that presented flaws in the adequacy of the partner’s treatment in relation to the pregnant woman’s treatment are highlighted in Figure 3.

## 4. Discussion 

The study showed that the diagnosis of maternal syphilis was predominant in the third trimester of pregnancy at 69%. Only 1.6% of the pregnant women were registered with an adequate treatment regimen; of these, 16.3 % had the concomitant treatment with their partners. Of the affected children, 78.8% were registered as asymptomatic, 73.5% had no record of performing the non-treponemal cerebrospinal fluid test, and 47% did not undergo long bone X-rays. The neighborhoods with greater syphilis involvement in pregnant women were also those with the highest percentage of maternal treatment, not concomitant with the partner. The highlights were the neighborhoods of Felipe Camarão and Quintas. Triggers for vertical transmission were the late diagnosis of the pregnant woman and the sexual partnership, in addition to the weaknesses in the clinical/therapeutic management of the disease.

The cumulative rate of attack or incidence for vertical transmission found in this study was higher (56.8%) than that found in other investigations, where rates ranged from 33.4% to 34.8% [9,10]. The high rates result from the low quality and effectiveness of the assistance to reduce the disease [9]. 

Regarding the groups of women notified, in this study, the results portray a social profile of vulnerability. It is noted that the same characteristics are described in the literature: women with less education [9,11], mixed-race [5,6], urban residents [12] and housewives [13]. A study [12] shows that inequalities in access and prenatal quality are factors that contribute to the greater exposure of children from less privileged classes to the risk of vertical transmission of CS. 

The most affected groups involve women between 20 and 35 years old [14,15]. Other investigations [16,17] correlate the incidence of syphilis with women who became sexually active before the age of 19, with multiple partners, and who exhibit sexual behaviors that are vulnerable to sexually transmitted infections (STIs). It is considered that this age group is more prone to risky sexual behavior [12]. 

Regarding the detection of the disease in the present investigation, it was observed that most were still identified prenatally; however, a significant number of cases were diagnosed late, especially in the third trimester. This data shows similar behavior in other Brazilian states [5,6]. However, a Brazilian epidemiological investigation [4] analyzing data between 2015 and 2016, observed a higher proportion of diagnoses in the first trimester (37%), which demonstrates an expansion of early diagnosis during prenatal care. 

Regarding prenatal pregnancy monitoring, it was found that although access to health services was recorded for all investigated cases, it was not an effective strategy for the treatment of cases and prevention of vertical transmission of syphilis. This shows that failures in care contribute to the persistence of congenital syphilis as a public health problem [9,12,13]. 

Regarding the diagnosis made at the time of delivery/postpartum, it should be noted that this is not considered the most opportune moment for the diagnosis, due to the risks of vertical transmission. In regard to this, a study [18] found that with each week of delayed treatment the risks of vertical transmission increase by 127%. Nevertheless, screening at the time of delivery is recommended, as it enables therapeutic follow-up before discharge and prevents further transmission of the disease [1]. 

Regarding the missed opportunities for screening for gestational syphilis, a study [19] conducted in the state of Ceará found that the difficulties faced by pregnant women to perform the VDRL exam are related to the difficult access to prenatal consultations, the limit in the collection of monthly exams, as well as queues and delays in getting the result. These data refer to the difficulty of access and inefficiency of the network services diagnostic support. 

The clinical form with the highest rates of fetal infection is primary syphilis [14], being the clinical type most identified in the present study. However, a majority of low titrations not consistent with this clinical staging of the disease were identified. The persistence of low titrations (1:4) of the non-treponemal or non-reagent test after positive treponemal testing is indicative of the presence of serologic scarring [20,21]. These may remain lifelong reagents in previously treated individuals. Serologic scarring, if there is a clinical history of appropriate treatment [20], is not a prerequisite for treatment and for reporting cases of gestational syphilis [22]. 

For cases where there are doubts as to the course of the disease, or latency with an unknown duration, treatment with three doses of benzathine penicillin is performed [1]. However, it is noteworthy that although most diagnoses were primary syphilis, the present investigation identified that the treatment profile for both the pregnant woman and the sexual partner was with three doses of benzathine penicillin. 

Regarding the completion of the notification form, most of it may have been performed in the wrong way, according to the epidemiological investigation [4]. The analysis of the information showed that according to the clinical classification, most cases were attributed as primary syphilis, not being in accordance with the pathophysiology of the disease [4]. Furthermore, a recent study [23] identified that health professionals had insufficient technical skills to address the problem of syphilis in prenatal care, especially regarding syphilis treatment and control [9,23,24,25]. 

The use of other therapeutic regimens for the treatment of pregnant women and their sexual partner(s) was found. however, only penicillin is an appropriate treatment for pregnant women; therefore, other treatments are considered inadequate. The use of ceftriaxone as an alternative treatment for pregnant women is recommended when penicillin treatment is impossible [1,26]. 

A factor of interest in the treatment of pregnant women in the period investigated refers to the problems in the supply of penicillin from the second half of 2014. This fact caused a delay in supply to states and municipalities due to a restricted demand and increased requests [5], which contributed to the expressed inadequacy in the treatment of the pregnant women investigated. 

The low adherence to treatment by the partner identified in this investigation is supported by data from the literature [6,12,13,23]. Difficulties in identifying partners and adhering to treatment may be related to working hours that are not compatible with the functioning of health services, lack of knowledge and the consequences of the disease [12]. 

One study listed the difficulties women face in communicating with their partner, namely: negative feelings, fear of death, betrayal and fear of prejudice [27]. In this way, adherence strategies for prenatal male involvement should be restructured, starting from the analysis of missed diagnostic and treatment opportunities, and also health education practices should be reinforced to ensure a healthy pregnancy without risks to the mother–child binomial [25]. 

Regarding the outcomes of syphilis for children, a predominance of asymptomatic cases and a low fatality rate of cases were identified when compared to another study [28]. However, there was a statistical correlation of symptoms when compared to VDRL titrations and maternal treatment. The likelihood of fetal infection is influenced by the stage of syphilis in the mother and the duration of fetal exposure. Thus, transmission is greater when women have primary or secondary syphilis during pregnancy and when there is longer exposure to the fetus [1,18].

The failures observed in the therapeutic follow-up in the delivery unit, such as the absence of other tests for the diagnostic screening of syphilis and the consequences of the injury to the child, were also detected in an investigation in Southeastern Brazil [6]. Identification of ascending titration should be performed following the non-treponemal test at 1, 3, 6, 12, and 18 months of age, with interruption of follow-up with two consecutive negative non-treponemal examinations and proceeding with the notification of this follow-up [1]. 

The notifications registered in the analyzed period point to a variation in the number of cases, with an emphasis on the year 2012 and a decline from 2013. These data corroborated with the statistics from other northeastern states, showing a decrease in cases [5]. It is noteworthy that in 2014, one of the criteria for case definition was changed, which may have impacted on the reduction of notifications. In this new rule, information that is ignored in regard to the treatment of the pregnant woman’s sexual partner is no longer considered a precept for reporting congenital syphilis [22]. However, reporting was continued for cases in which non-treatment of the partner(s) was reported [5]. 

As evidenced in this study and in the Brazilian literature [10,14,29,30], notification of syphilis presents weaknesses in surveillance mechanisms, with significant quantitative under-notification. There was a predominance of notifications in hospitals rather than basic health units, data corroborated by studies that suggest underreporting of cases at the primary care level [10]. 

The high number of missing data—either ignored or underreported—identified in this study, reflects a low valuation and priority regarding compulsory notification in the work routine and can be considered as an indirect evaluation of poor quality prenatal care [10,19,29]. 

It is suggested that there are unsatisfactory guidelines when clarifying the proper completion of all fields of the form, and that the structure of preparation of professionals for notification needs to be reorganized [10,14,23].

There has been a clear improvement in the intervals between the diagnosis and the notifications identified in the records of congenital syphilis and the relationship with the existence of HESNs in the institutions of delivery care. This reality represents an instrumental knowledge of the professional in the nucleus in regard to the work processes of epidemiological surveillance and the value of the transfer of information of the illness to the health authorities. 

The geo-referencing of the diseases allowed us to identify an intensification of cases in locations of greater social vulnerability and low economic condition in the urban area of the city, which demonstrates the need to plan strategies to improve access and prenatal care in neighborhoods at risk. The authors point to the need for an in-depth study of the social determinants of syphilis in this spatial scope [31]. 

In addressing the limitations of this study, we emphasize the restriction of information from secondary data, with the possibility of data loss and bias due to underreporting. Moreover, as this is a retrospective study, the possibility of data loss remains. Finally, the quality of data filling, with the amount of data ignored, makes it difficult to collect all information, thus emphasizing the importance of proper file filling as essential to improving the care provided to those involved.

Nonetheless, we emphasize that it is from these notifications that the monitoring of the diseases and the planning of the actions taken are organized through decision making in the various spheres of health management, which strengthens the data of this investigation by analytically aiding the reality of notification of maternal and congenital syphilis, being the object of study of this investigation. The reality posed in this study is believed to prompt prospective and evaluative studies to complement the information necessary for effective decision-making in controlling maternal and congenital syphilis. 

## 5. Conclusions

Focusing on the notifications of gestational and congenital syphilis showed the ineffectiveness of the methods necessary to control the disease for the population in this research. The factors that trigger vertical transmission are related to the late diagnosis of the pregnant woman and the type of sexual partnership, as well as the weaknesses in the clinical/therapeutic management in relation to the phase of the disease, all being factors that contributed to a high rate of 56.8% probability of vertical transmission. Detection of the disease was predominantly during prenatal care, especially in the third trimester. It was also found that access to health services, recorded in all investigated cases, was not an effective strategy for the treatment of cases and in the prevention of vertical transmission of syphilis, suggesting failure in the process of diagnosis, treatment and control, especially in primary health care services. 

We highlight the inability to ensure the continuity of maternal treatment and treatment for the sexual partner(s), as well as the use of medications recommended for the treatment of pregnant women. The low adherence of the partner to the treatment of syphilis identified in the sample is significant, as it corroborates with reinfections and the continued spread of the disease to other sexual partners. 

Currently, programs are available from the Unified Health System aimed at men’s health, such as Family Planning, Partner’s Prenatal Program, National Policy for Integral Attention to Men’s Health, and rapid tests for syphilis screening in the units of health.

Thus, it is suggested in the places with the most significant number of cases, the strategic implementation of screening for the detection of the disease in men and women of reproductive age, aiming to treat it early. It is suggested that there should be an increase in the offer of times to perform the rapid test in the primary health units, as well as health education actions with the group and age group most exposed to the risk of acquiring the disease, also the effective implementation of the Partner’s Prenatal.

In addition, appropriate training of health professionals to deal with subjective demand at the time of diagnosis is suggested. Issues related to risky sexual behavior of sexual partners, infidelity, the possibility of divorce, or fear of communicating the diagnosis of STD to the partner, are involved in this. Finally, it is suggested to make effective and viable the administration of Penicillin in the primary units. Thus, ensuring higher follow up of these patients without the need to move these patients to other health facilities in order to receive treatment for syphilis.

Regarding the outcomes of congenital syphilis, we note the predominance of treatment of newborns still in the hospital unit. However, the occurrence of failures in the clinical evaluation and identification of sequelae is evident due to the low registering of long bone X-rays and Cerebral Spinal fluid collection. 

Notification records revealed a lack of important information, whether from ignored or under-reported data. Also noteworthy is the improvement in notification processes when carried out from the Hospital Epidemiological Surveillance Centers. Strategies for professional training in reporting and expanding the provision of information to epidemiological surveillance centers are essential for the continuation of the analysis of the disease and the strengthening of program actions to control the disease. 

Finally, it is noteworthy that the analysis of this study is based on the implementation period of the Cegonha (Stork) Network in 2011, continuing until December 2015, the final date for consolidation of the Multi-annual Plan (2012–2015) to eliminate congenital syphilis cases. In this context, there is a low effectiveness of these Health Care Policies in preventing vertical transmission of syphilis to the population studied.

We emphasize the need for early screening of sexually transmitted infections for men and women of childbearing age, establishment of methods of adherence of sexual partners to treatment and clinical follow-up, as well as uniformity in the follow-up of therapeutic protocols by the health technicians. 

It is suggested that a case-by-case monitoring network be established to consolidate effective prenatal care that ensures a safe birth, free from harmful occurrences of the binomial, especially in regard to syphilis, a preventable and notoriously neglected condition. 

## Figures and Tables

**Table 1:** Time of diagnosis and laboratory aspects of detection of gestational syphilis. Natal, RN, Brazil, 2016 (*n* = 129 pregnant women).

Variable	*N*	%
Diagnoses of maternal syphilis		
1st Trimester	10	7.8
2nd Trimester	28	21.7
3rd Trimester	89	69.0
Gestational age Ignored	2	1.6
Total	129	100
Confirmatory Treponemal Test		
Reagent	24	18.6
No reagente	4	3.1
Not accomplished	87	67.4
Ignored	14	10.9
Total	129	100
VDRL titration of pregnant woman		
1 to 8	51	39.5
12 to 16	25	19.4
32 to 64	36	27.9
Above 64	3	2.3
Losses *	14	10.9
Total	129	100

Note: (VDRL) Venereal Disease Research Laboratory; * Losses—Blank fields, not completed on the notification form.

**Table 2:** Diagnosis of maternal syphilis according to sociodemographic variables, using Chi-Square and Fisher tests with a significance level of 5%. Natal, RN, Brazil, 2016 (*n* = 129).

Sociodemographic Variables	Diagnosis of Maternal Syphilis			*p*
	At Prenatal Care *N* (%)	Delivery/Postpartum *N* (%)	Total *N* (%)	
Age				0.418
14 to 20 years	19 (59.4)	13 (40.6)	32 (25.8)	
20 to 35 years	40 (51.3)	38 (48.7)	78 (62.9)	
Over 35 years	9 (64.3)	4 (28.6)	14 (11.3)	
Race				0.720
White	16 (64.0)	9 (36.0)	25 (17.6)	
Black	3 (60.0)	2 (40.0)	5 (4.2)	
Mixed Race	49 (54.4)	40 (44.6)	89 (74.8)	
Educational level				0.754
Illiterate	0 (0.0)	1 (100.0)	1 (1.2)	
Elementary School	38 (53.5)	32 (46.5)	70 (84.3)	
High School	6 (50.0)	6 (50.0)	12 (14.5)	

Note: (*p*) *p*-value.

**Table 3:** Relationship between the titles of the diagnostic tests of gestational and congenital syphilis with signs and symptoms. Natal, RN, Brazil, 2016 (*n* = 101 pregnant women, *n* = 73 children).

Variable	Sign or Symptoms	*N*	%	Mean	Standard Deviation	Student’s *t*-Test	Confidence Interval (95%)	*p*
Maternal VDRL	Yes	10	9.9	17.2	20.0	8.246	[15.58; 25.37]	<0.001 *
	No	91	90.1	22.9	25.5			
Child VDRL	Yes	8	10.9	8.1	10.9	4.537	[2.806; 7.139]	<0.001 **
	No	65	89.0	6.7	9.2			

Note: (L) Losses, without information; (*p*) *p*-value; (VDRL) Veneral Disease Research Laboratory; * <0.001—Assumes equal variances, use of Bartlett’s test with *p*-value 0.371; ** <0.001—Assumes equal variances, use of Bartlett’s test with *p*-value 0.536.
